# Habitual water intake impacted the body composition of young male athletes in free-living conditions: a cross-sectional study

**DOI:** 10.3389/fspor.2024.1458242

**Published:** 2024-10-22

**Authors:** Jianfen Zhang, Na Zhang, Yibin Li, Hairong He, Ge Song, Junying Chen, Yi Yan, Guansheng Ma

**Affiliations:** ^1^ Department of Nutrition and Food Hygiene, School of Public Health, Peking University, Beijing, Haidian, China; ^2^ Department of Student Nutrition, National Institute for Nutrition and Health, Chinese Center for Disease Control and Prevention, Beijing, Xicheng, China; ^3^ Laboratory of Toxicological Research and Risk Assessment for Food Safety, Peking University, Beijing, Haidian, China; ^4^ Institute for Nutrition and Food Hygiene, Beijing Center for Disease Prevention and Control, Beijing, Dongcheng, China; ^5^ Department of Sport Biochemistry, School of Sport Science, Beijing Sport University, Beijing, Haidian, China; ^6^ Guangdong Ersha Sports Training Center, Guangzhou, China

**Keywords:** body composition, total water intake, total drinking fluids, association, hydration status, young athletes

## Abstract

The study aimed to explore the associations between water intake and body composition and differences of body composition in different water itake and hydration statuses among young male athletes. A cross-sectional study was conducted among 111 young male athletes in Beijing, China. Total drinking fluids (TDF) and water from food were assessed using a 7-day, 24-h fluid intake record questionnaire and the duplicate portion method, respectively. The osmolality of 24-hour urine and blood samples was tested. Body composition was measured using a bioelectrical impedance analyzer twice at 5-min intervals. Participants were divided into two groups based on the recommendations of total water intake (TWI) and TDF in China, as well as into three groups based on 24-h urine osmolality. Pearson's correlation coefficients were calculated to determine the relationship between water intake and body composition. Chi-square tests and Student's *t*-tests were used to compare differences. A total of 109 participants completed the study. TDF (*r* = 0.230, *p* = 0.016; *r* = 0.234, *p* = 0.014; *r* = 0.242, *p* = 0.011) and TWI (*r* = 0.275, *p* = 0.004; *r* = 0.243, *p* = 0.011; *r* = 0.243, *p* = 0.011) were positively correlated with total body water (TBW), intracellular water (ICW), and extracellular water (ECW). TBW/body weight (BW) was positively associated with TDF percentage of BW (TDF/BW) (*r* = 0.267, *p* = 0.005), water from food percentage of BW (*r* = 0.217, *p* = 0.024), and TWI percentage of BW (TWI/BW) (*r* = 0.316, *p* = 0.001). Participants who met the TDF recommendation of China had 1.3 kg higher skeletal muscle mass (SMM), 0.9 kg higher ICW, and 0.5% higher TBW/BW than those who did not (all *p* < 0.05), with fat-free mass (FFM) and TBW being higher (*p* = 0.051; *p* = 0.050). Those who met the TWI recommendation of China had 1.3 kg higher SMM, 2.4 kg higher FFM, 1.1 kg higher ICW, 0.6 kg higher ECW, and 1.7 kg higher TBW than their counterparts (all *p* < 0.05). Moderate associations were found between water intake and body composition. No significant differences were observed among participants in three hydration statuses (all *p* > 0.05). Participants who met the TWI or TDF recommendations had better body composition distribution than their counterparts. Thus, habitual water intake, not hydration status, affects body composition among athletes in free-living conditions.

## 1 Introduction

Water is essential for life. Within the human body, water is distributed in intracellular and extracellular compartments. Intracellular water (ICW), which represents 60% of total body water (TBW), is the main determinant of cell volume. Extracellular water (ECW), which represents the other 40% of TBW, includes plasma, interstitial fluid, and other transcellular fluids such as cerebrospinal fluid, synovial fluid, and vitreous body fluid (1). Without water, humans can survive only for a few days. The human body obtains water from total drinking fluids (TDF), water from food, and metabolic water. TDF and food each contribute about 50% of total water intake (TWI), whereas metabolic water represents about 250–350 ml/day (2, 3). Therefore, water intake is important for the distribution of body water.

There is a dynamic balance between water intake and water output. When water intake is roughly equal to the output, people are in an optimal hydration status. Otherwise, individuals may experience hypohydration when the water intake does not compensate for water losses. A large body of research has reported that dehydration induces deficits in cognitive performance among adults (4–6). Even mild hypohydration is associated with an increased prevalence of obesity, insulin resistance, diabetes, and metabolic syndrome (7–9). Nevertheless, the evidence was regarded the non-athletic population, for athletes, the situations of hypohydration on health maybe more serious. It is accepted that the core body temperature increases during exercise, and blood flow to the skin increases concurrently to remove heat through sweat. Therefore, sweat loss during exercise that exceeds water intake may lead to dehydration among athletes (10). Additionally, a study conducted among mice showed that muscle is the first organ to lose water; thus, fluctuations in hydration status may directly affect muscle function (11). In this sence, evidence of effects of hydration status in neuromuscular function has been collected among athletes (12, 13). Indeed, dehydration over 2% of body weight impairs endurance exercise performance (14), including endurance cycling performance (15), and maximal aerobic capacity (16), by reducing blood volume, muscle blood flow, and thermoregulation (17). Moreover, that a deficit in body water may also impacted the performance during exercise through an increase in mood disturbance and subjective discomfort (18, 19), and may contribute to the decreased in endurance performance with hypohydration (20). Although strategies to maintain proper hydration status for athletes have been proposed (21–26), a substantial number of studies (27–31) indicate that a significant percentage of athletes are undehydrated before, during, and after exercise. Future research should help raise awareness of hydration status among athletes.

Studies have revealed a positive association between water intake and body water content (32–35). Individuals with different levels of water intake show different hydration biomarkers, including urine osmolality and urine specific gravity (USG), among young adults and athletes (36–39). Furthermore, young adults who meet the total fluids intake recommendation of China have higher ICW, ECW, and TBW than those who do not, in free-living conditions (32). Moreover, relative water turnover in participants with active physical activities is significantly greater than that in the sedentary group (40). Body composition may differ between athletes and non-athletes among adults. However, in China, few studies have evaluated the body composition of young adults (32, 41–43) and even fewer have focused on athletes or explored the associations between water intake and body composition, which needs more attention. Additionally, a large proportion of athletes in China are not in euhydration status (44, 45), which may cause deleterious effects on performance, especially in hot weather. To date, differences in body composition among athletes with different hydration statuses have not been investigated.

Monitoring body composition, including TBW, ICW, and ECW, may represent an additional and valuable approach to control for potential body composition changes linked to physical performance in athletes. TBW and ICW are closely correlated with muscle mass, both in male and female elderly people, indicating that those with higher ICW have better functional performance and lower frailty risk (46). Furthermore, a reduction of 3%–4% in TBW caused by dehydration would attenuate muscular strength and power. ICW determines cell volume and is believed to affect metabolism, as water impacts protein structure and enzymatic activity (47, 48). Moreover, ICW is a good predictor of strength and power in athletes (11, 49), with decreases in ICW correlated with impaired muscle strength. Additionally, the ECW/ICW ratio can predict knee extension force and gait speed, unaffected by age and sex (50). Therefore, considering the importance of body composition for athletes, frequent investigations should take place. Although scientific studies investigating the body composition of athletes are growing, they are still limited. Therefore, more studies need to be initiated.

This study aimed to explore the associations between water intake and body composition and investigate differences in body composition among young athletes who meet the recommendation of TWI or TDF or not. Furthermore, we also aimed to compare the values of body water compartments among participants with different hydration statuses. With this research, we aimed to contribute to the provision of a science-based education on fluid intake for young adults.

## 2 Methods

### 2.1 Participants

A cross-sectional study was designed, and 111 young adult males were recruited in Beijing, China. The inclusion criteria were as follows: healthy adult male college students aged 18–25 years with a regular exercise training plan (more than five moderate-intensity exercises per week). The exclusion criteria were as follows: those with chronic diseases such as oral cavity (including the mouth ulcer or chronic cheilitis), endocrine, kidney, gastrointestinal tract, and metabolic diseases; sports injuries; cognitive impairment; and those who have taken drugs, vitamins, or other health products within the past month. The study protocol was approved by the Peking University Institutional Review Committee. The ethical approval project identification code is IRB00001052-19051. The study protocol has been registered on the Chinese clinical trial registry website, and the identification code is Chi CTR 1900025710. This study was conducted according to the guidelines of the Declaration of Helsinki. All subjects signed an informed consent form before participating in the study.

### 2.2 Sample size calculation

In a similar study (51), more than 75% of the random urine samples of 4.4% of the participants were of optimal hydration status, with the set *t* = 1.96 (*α* = 0.05), *e* = 4%. Thus, according to the formula for calculating the sample size of simple random sampling, *n* = t^2^p (1-p)/e^2^, the maximum sample size is 101. Considering the dropout rate, set at 10%, 111 participants were needed for this research.

### 2.3 Study procedure

**Figure 1:**
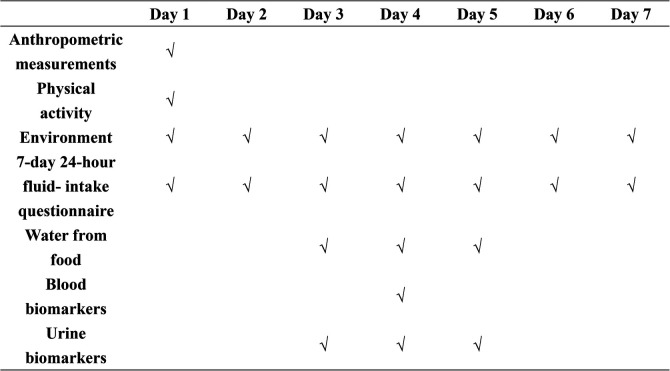
Study procedure.

A total of 111 participants were recruited, and 109 of them completed the study, totalizing a completion rate of 98.2%. The athletes were all classified at least as tear 3 according to Mckay and colleagues (52). The study spanned 7 consecutive days, including 5 weekdays and 2 weekends. On the first study day, anthropometric measurements, including height, weight, and waist circumference, were taken. For recording fluid intake from water and other beverages, all participants were instructed several times on how to record the related information on the self-designed 7-day, 24-h fluid intake questionnaire. In addition, over the 7 consecutive days, all the foods that the participants ate were weighed and recorded for 3 consecutive days (2 weekdays and 1 weekend day, from day 3 to day 5). Furthermore, during these 3 days, 24-h urine samples, including the first morning urine, were collected by the participants. On day 4, the fasting venous blood samples of all participants were collected. Moreover, the indoor and outdoor temperature and humidity were recorded each day for 7 days. The study procedure is shown in Figure 1.

### 2.4 Measurement of TWI

A 7-day, 24-h fluid intake record questionnaire was used to assess the TDF (53–55), which was designed by the investigator, as described previously (36). Participants were asked to complete the questionnaire for 7 consecutive days (5 weekdays and 2 weekends). The type and amount of fluid intake for each time were measured by a standard cup provided by the investigator. Furthermore, to ensure completeness and accuracy, the investigators checked the questionnaire every day. Before filling the records, a certified researcher provided instructions about how to recorded fluids intake each time. Furthermore, in order to avoid forgetting to record information about drinking water, notices were sent through WeChat, an instant messaging apps widely used by college students, to remind them to record (Tencent Holdings Ltd., Shenzhen, China).

All foods that the participants ate for the 3 days were weighed before and after eating, to calculate the amount of water from food (YP20001, SPC, Shanghai, China). Water from food was assessed using the duplicate portion method. Moreover, all food samples were collected by the investigators and sent to the laboratory for immediate storage. All food samples were measured according to the national standard GB 5009.3-2016, and the water from fruits or other snacks was assessed according to the China Food Composition Table (2016). The water from food was separated into five categories, as described in our previous study (36). Before three days of foods intake survey, participants were showed how to eat the foods, such as how to place chicken bones or fish bones, etc.

### 2.5 Temperature and humidity of the environment

The indoor and outdoor temperature and humidity were recorded each day for 7 days (WSB-1-H2, Exasace, Zhengzhou, China). The average temperature was 24.2°C ± 5.8°C at 10 a.m. each day, and the average humidity was 29.5% ± 15.8% RH during the study.

### 2.6 Anthropometric measurements

Height, weight, and waist circumference were measured twice by trained investigators using standard procedures in the morning of the first day of the study, with height was measured twice to the nearest 0.1 cm in bare feet and light clothes and fasting body weight (participants were asked to voided) was measured twice to the nearest 0.1 kg (HD2M-300; Huaju, Zhejiang, China; Accu Measure, Greenwood Village, CO, USA). Averages for height and weight were calculated. BMI was calculated as weight (kg)/height squared (m²).

The body composition was tested by a trained investigator using a bioelectrical impedance analyzer in the fifth day and seventh day of the study days (BIA; Inbody 230, Inbody, Seoul, Korea). Fasting body composition was evaluated with participants were asked to voided and with light clothes and bare feet. Each participant was tested twice. Before being tested, participants were asked not to eat or drink anything and to remain quiet for at least 15 min. The researcher entered the participant's number, age, and sex into the BIA, and the participants stood on the foot electrodes while holding the hand electrodes tightly. The test lasted about 3 min. During the test, participants were asked to stand still with an angle of about 15° between their trunk and upper limbs. After the data were displayed on the BIA, the participant stepped off the instrument and then stood on the foot electrodes again for the second measurement. The value used was the average of the two examinations.

### 2.7 Urine and plasma biomarkers

During the 3 consecutive days (2 weekdays and 1 weekend, Thursday, Friday, and Saturday), which was from the fifth day to the seventh day of the study days, participants were asked to use customized urine collection bags to collect all random urine and filled out the urination behavior questionnaire, recording the time and volume of each urination. Furthermore, all the urine samples were collected, except the first urine samples on the morning of the fifth day. The collected urine was immediately sent to the laboratory. Trained investigators weighed the collected urine (YP20001, SPC, Shanghai, China), recorded and sampled it, and then stored the urine in a refrigerator at +4℃. In order to avoid the mistakes in recording urine, there were three actions. Firstly, participants should record the related information of each urine on the questionnaire and the urine collection bags; secondly, investigators were asked to recorded each urine the participants sent to the lab; thirdly, the amounts of the TWI and the volume of the urine would be checked. If there were some mistakes in the collection of the urine, the investigators would report to the researchers to find solutions.

Urine osmolality, USG, pH, urea, creatinine, and urine electrolyte concentrations (including sodium, potassium, chloride, calcium, magnesium, and phosphate) were tested using standard procedures (45). Fasting venous blood samples were collected by trained investigators to measure the concentrations of Na, K, Cl, testosterone, cortisol, creatinine, and copeptin, as described in our previous study (45). Osmolality of urine samples and plasma were assessed with freezing point method by osmotic pressure molar concentration meter (SMC 30C; Tianhe, Tianjin, China). USG, pH, urea and creatinine were tested by automatic urinary sediment analyzer with uric dry-chemistry method (H-800; Dirui, Changchun, China). The electrolyte concentrations of urine and plasma samples (including sodium, potassium, chloride, calcium, magnesium and phosphate) were evaluated by automatic biochemical analyzer with the ion-selective electrode potentiometer method (AU 5800; Beckman, Brea, CA, USA). The testosterone, cortisol, creatinine were determined by a trained investigator using Imark microplate reader (Bio-Rad 680, Bio-Rad, Hercules, CA, USA). The creatinine level was assessed using the sarcosine oxidase method (C011-2-1, Jiancheng, Nanjing, China).

Participants were divided into three groups: optimal hydration status (defined as ≤500 mOsm/kg), medium hydration status (defined as 500 mOsm/kg <urine osmolality ≤800 mOsm/kg), and dehydration [defined as urine osmolality >800 mOsm/kg (1, 52, 56, 57)]. Moreover, participants were also assigned to two groups: those met the recommendation of TWI of China (≥AI), and those failed to meet the recommendation of TWI of China (<AI). Finally, participants were split into two groups: those met the recommendation of TDF of China (≥AI), and those failed to meet the recommendation of TDF of China (<AI).

### 2.8 Statistics

SAS 9.2 software (SAS Institute Inc., Cary, NC, USA) was used for statistical analysis. Data are presented as mean ± standard deviation or median and interquartile ranges if the data were not normally distributed. Chi-square tests and Student's *t*-tests were used to compare differences among groups. Pearson's correlation coefficients were calculated to determine the relationship between water intake and body composition. The significance level was set at 0.05.

## 3 Results

Table 1 shows the characteristics of the participants. Age, height, weight, BMI, and skeletal muscle of the participants were 20.8 years, 178.7 cm, 70.7 kg, 22.1 kg, and 34.9 kg, respectively (Supplementary Table S1). The amounts of TWI, TDF and water from food are displayed in Supplementary Table S2. The amounts of the TWI, TDF and water from food were 2,701 ml, 1,789 ml and 955 ml, respectively; with TDF contributing about 65.0% to TWI. Furthermore, the main contributor of TDF was water, which was 1,181 ml; while, the intake of sugar sweetened beverage was 469 ml.

**Table 1:** Body composition of participants who met or did not meet the adequate intake of TWI and TDF of China.

Variable	TWI/China				TDF/China				Total
	≥AI	<AI	*F*	*p*	≥AI	<AI	*F*	*p*	
SMM	35.7 ± 4.0^#^	34.4 ± 2.6	−2.186	0.031	35.4 ± 3.8*	34.2 ± 2.4	−1.990	0.049	34.9 ± 3.3
FFM	62.7 ± 6.7^#^	60.3 ± 4.2	−2.258	0.026	62.2 ± 6.2	60.1 ± 4.0	−1.974	0.051	61.3 ± 5.4
ICW	29.0 ± 3.1^#^	27.9 ± 2.0	−2.241	0.027	28.7 ± 2.9*	27.8 ± 1.8	−2.016	0.046	28.3 ± 2.5
ECW	16.9 ± 1.8^#^	16.3 ± 1.1	−2.247	0.027	16.8 ± 1.7*	16.3 ± 1.1	−1.913	0.058	16.5 ± 1.5
TBW	45.9 ± 4.9^#^	44.2 ± 3.1	−2.251	0.026	45.5 ± 4.5*	44.0 ± 2.9	−1.986	0.050	44.8 ± 3.9
BW	72.3 ± 8.8	70.2 ± 6.1	−1.473	0.144	71.9 ± 8.4	70.0 ± 5.7	−1.326	0.188	71.0 ± 7.3
ICW/FFM (%)	46.2 ± 0.2	46.2 ± 0.2	0.351	0.726	46.2 ± 0.3	46.2 ± 0.2	−0.514	0.609	46.2 ± 0.3
ECW/FFM (%)	27.0 ± 0.3	27.0 ± 0.3	0.062	0.951	27.0 ± 0.3	27.0 ± 0.3	0.392	0.696	27.0 ± 0.3
ICW/TBW (%)	63.1 ± 0.4	63.1 ± 0.3	0.073	0.942	63.1 ± 0.4	63.1 ± 0.3	−0.447	0.656	63.1 ± 0.4
ECW/TBW (%)	36.9 ± 0.4	36.9 ± 0.3	−0.082	0.934	36.9 ± 0.4	36.9 ± 0.3	0.457	0.649	36.9 ± 0.4
ECW/ICW (%)	58.5 ± 1.0	58.5 ± 0.8	−0.092	0.927	58.5 ± 0.9	58.4 ± 0.9	0.447	0.656	58.5 ± 0.9
TBW/BW (%)	63.6 ± 2.8	63.1 ± 3.2	−0.890	0.375	63.5 ± 3.1	63.0 ± 3.1	−1.990	0.049	63.3 ± 3.1
TBW/FFM (%)	73.2 ± 0.2	73.2 ± 0.1	0.692	0.490	73.2 ± 0.2	73.2 ± 0.1	−0.131	0.896	73.2 ± 0.2

Note: Values are shown as mean ± standard deviation (SD).

*Significant difference was found between the group ≥AI of TDF/China and <AI of TDF/China (*p* < 0.05).

^#^Significant difference was found between the group ≥AI of TWI/China and <AI of TWI/China (*p* < 0.05).

The urinary and plasma biomarkers of the participants are showed in Supplementary Tables S3, S4. The volume, osmolality, specific gravity, pH and the concentrations Na, K and Cl of 24 h urine were 850 ml, 764 mOsm/kg, 1.020, 6.3, 202 mmol/L, 45.21 mmol/L and 221 mmol/L, respectively. Almost about of the participants were hypohydrated only 15.6% of them were in optimal hydration status.

### 3.1 Intracellular and extracellular fluid and fluid intake of the participants

Table 1 presents the differences in the mean body composition of participants who met the recommended adequate intake of water or not. Participants who met the recommendation for TDF in China had higher skeletal muscle mass (SMM), fat-free mass (FFM), ICW, and TBW/body weight (BW) (all *p* < 0.05), with TBW and ECW tending to be significantly different (*p* = 0.050; *p* = 0.059), when comparing with those falied meeting the recommendation. Similarly, those who met the recommendation for TWI in China had higher SMM, FFM, ICW, ECW, and TBW than their counterparts (all *p* < 0.05). It should be noted that the values of SMM, FFM, ICW, ECW, and TBW were all assessed by BIA in the current study.

### 3.2 Correlation between intracellular and extracellular fluid and fluid intake

Table 2 shows that TDF and TWI were positively correlated with TBW, respectively (*r* = 0.232, *p* = 0.015; *r* = 0.275, *p* = 0.004); simultaneously, significant associations were found between TDF and TWI with ICW, respectively (*r* = 0.234, *p* = 0.014; *r* = 0.243, *p* = 0.011); TDF and TWI were significantly associated with ECW, respectively (*r* = 0.243, *p* = 0.011; *r* = 0.242, *p* = 0.011). A significant correlation was also found only between ICW and water from food (*r* = 0.199, *p* = 0.038), but no significant associations were found between ECW and water from food (*r* = 0.161, *p* = 0.094), TBW and water from food, respectively (*r* = 0.185, *p* = 0.054).

**Table 2:** Correlation between water intake normalized by body weight from all the sources analyzed with anthropometric and body composition variables of males.

Variable	TDF/weight (ml/kg)		Water from food/weight (ml/kg)		TWI/weight (ml/kg)	
	*r*	*p*	*r*	*p*	*r*	*p*
BW	−0.177	0.065	−0.194	0.043	−0.231	0.016
FFM	−0.061	0.526	−0.102	0.291	−0.095	0.326
FM	−0.264	0.006	−0.237	0.013	−0.321	0.001
TBW	−0.058	0.549	−0.103	0.288	−0.092	0.339
TBW/BW	0.267	0.005	0.217	0.024	0.316	0.001
ICW	−0.009	0.928	−0.078	0.423	−0.076	0.430
ECW	−0.007	0.945	−0.105	0.279	−0.080	0.407

Note: Positive correlations were found for TDF and TWI with TBW, ICW and ECW, respectively (*r* = 0.232, *p* = 0.015; *r* = 0.275, *p* = 0.004; *r* = 0.230, *p* = 0.016; *r* = 0.279, *p* = 0.003; *r* = 0.233, *p* = 0.015; *r* = 0.265, *p* = 0.005); moreover, significant correlation was found between ICW and water from food (*r* = 0.199, *p* = 0.038), but no significant differences were found for ECW and TBW with water from food, respectively (*r* = 0.161, *p* = 0.094; *r* = 0.185, *p* = 0.054).

### 3.3 Body composition of participants with different hydration statuses

No statistically significant differences were noted in SMM, FFM, ICW, ECW, TBW, BW, ICW/FFM, ECW/FFM, ICW/TBW, ECW/TBW, ECW/ICW, TBW/BW, and TBW/FFM among participants in the three hydration groups (*p* > 0.05) (Table 3).

**Table 3:** Body composition of participants with different hydration statuses.

Variable	Optimal hydration	Medium hydration	Dehydration	*F*	*p*
SMM	34.0 ± 2.8	35.1 ± 3.4	35.0 ± 3.3	0.733	0.483
FFM	60.0 ± 4.8	61.6 ± 5.6	61.4 ± 5.5	0.605	0.548
ICW	27.6 ± 2.2	28.5 ± 2.6	28.4 ± 2.5	0.736	0.481
ECW	16.3 ± 1.3	16.6 ± 1.5	16.6 ± 1.5	0.376	0.688
TBW	43.9 ± 3.5	45.1 ± 4.1	45.0 ± 4.0	0.590	0.556
BW	69.9 ± 6.9	71.5 ± 8.1	71.0 ± 6.8	0.286	0.752
ICW/FFM (%)	46.1 ± 0.3	46.2 ± 0.2	46.2 ± 0.2	1.534	0.220
ECW/FFM (%)	58.8 ± 1.1	58.3 ± 0.9	58.5 ± 0.8	2.307	0.105
ICW/TBW (%)	63.0 ± 0.4	63.2 ± 0.4	63.1 ± 0.3	2.169	0.119
ECW/TBW (%)	37.0 ± 0.4	36.8 ± 0.4	36.9 ± 0.3	2.191	0.116
ECW/ICW (%)	58.8 ± 1.1	58.3 ± 0.9	58.5 ± 0.8	2.194	0.116
TBW/BW (%)	63.0 ± 2.4	63.3 ± 3.6	63.5 ± 2.8	0.143	0.867
TBW/FFM (%)	73.2 ± 0.2	73.2 ± 0.2	73.2 ± 0.1	0.616	0.542

Note: Values are shown as mean ± standard deviation (SD).

## 4 Discussion

The present study is the first to investigate differences among athletes with different water intakes and hydration status and to evaluate the relationship between water intake and body composition in China. The main findings of the current study were threefold: (1) moderate associations between water intake and body composition; (2) young male athletes with adequate water intake had a significant differences in body water; (3) participants in different hydration statuses had similar body water.

It is worth noting that the body composition of the participants were assessed by BIA. BIA is a technology that quantitatively measures body composition through impedance that occurs when an electric current flows through the human body. The instrument measuring the body composition in this study utilizes the molecular method to quantify four body components of body water, protein, minerals, and body fat. The instrument uses 8-point contact electrodes (two thumb electrodes, two palm electrodes, two sole electrodes and two heel electrodes) to measure 30 impedance values at 5 segments (left and right upper limbs, trunk and lower limbs) at 6 different frequencies (1 kHz, 5 KHz, 50 KHz, 250 kHz, 500 KHz and 1,000 kHz), so as to accurately analyze the total water content. BIA has been widely used to quantify body composition, including ICW, ECW, and TBW of athletes (58–63), and has been compared with densitometry and dilution techniques as references (60, 64, 65).

In this study, ICW, ECW, and TBW were found to be 28.3 kg, 16.5 kg, and 44.8 kg, respectively, with TBW accounted for 63.3% of BW. In a study conducted among the young general population (32), TBW, ICW, and ECW were reported as 32.8 kg, 20.5 kg, and 12.4 kg, respectively, which were 12.0 kg, 7.8 kg, and 4.1 kg lower than the values reported in the current study, indicating that the body composition of athletes differ from that of the general population; these findings are consistent with those of a study conducted in Japan, in which the TBW of athletes was 3.2 kg higher than that of non-athletes (66). Furthermore, previous work has reported differences in ECW/TBW between athletic and non-athletic children (67). The results of the study confirmed differences between athletes and the general population. Additionally, due to differences in sweat output during increased energy expenditure from physical activity, the contribution of dietary composition to water turnover rates differs between active and sedentary men (40). In another study, TBW, ICW, and ECW among athletes were 47.46 kg, 28.86 kg, and 18.60 kg, respectively, which were all higher than the values reported in the present study, even though participants were of the same age (63). This may be attributed to racial and ethnic differences (68). Furthermore, FFM hydration (73.3%) reported in the present study was similar to that reported in a previous study (66).

Optimal body composition plays a significant role in athletic performance (69). ICW determines cell volume and impacts cell metabolism, and its depletion impairs the availability of nutrients and may produce intracellular catabolic effects (47, 49). ICW is an indicator of muscle quality and cell hydration and is related to muscle strength and functionality (70, 71). In the present study, participants who met or exceeded the recommendations for TWI had higher SMM, FFM, ICW, ECW, and TBW than their counterparts. Furthermore, those who met the recommendation for TDF in China had higher SMM and ICW than those who did not. A similar trend was found, including the ECW/TWI and TBW/BW, among general young adults aged 18–23 years in a previous study (32). In addition, a finding in agreement with other studies in the literature was that our present results confirmed moderate associations between water intake and body composition among athletes in China. Hence, those who achieved the adequate intake of TWI had better distribution of body composition and may also have better physical performance than those who did not. These results also indicated that water intake could impact the distribution of body composition among athletes. Nevertheless, it has been reported that LOW athletes exhibit higher USG when comparing with those with higher TWI; while TBW, ICW, ECW, and FFM hydration were not statistically different between groups (39). The population composition and methodologies in the two studies explained the differences of results. The study included males and females; the body water as measured by dilution techniques; the USG was the diagnosis of hydration status, and the participants were split into two groups with their habitual TWI obtained by seven-day food records, according to the standard of EFSA. While, in the present study, only young male athletes were included; BIA was used to assess the body water; the hydration status was evaluated by the osmolality of 24 h urine; the TWI was measured by questionnaire for 7 consecutive days of TDF survey (measured by a cup to the nearest 5 ml and recorded fluids intake each time) and 3 days of water from food survey (duplicate portion method) and participants were divided according to the recommendation of TWI or TDF China. Further studies were needed to explore this issue.

Regarding to the TDF and hydration status, in a previous study, a higher proportion of participants with higher habitual TDF had optimal hydration status than their counterparts (36). Thus, those with higher habitual TDF may have a lower risk of being dehydrated. Especially, the current study presents the need for athletes to maintain adequate water intake and the importance of educating athletes on proper hydration.

As previously mentioned, hydration is important for athletes, and hypohydration, mainly as a result of sweat loss among athletes, impairs physical performance, particularly reducing endurance performance (12, 13, 72, 73). Furthermore, hydration status also affects body composition. In our study, ICW, ECW, and TBW among athletes with different hydration statuses did not differ, similar to the results of another study (74). The study exploring the water compartments of athletes with differing hydration statuses demonstrated that ICW, ECW, TBW, and other variables of body composition did not differ between different hydration groups (74). Notwithstanding, in some acute heat-induced or water restriction-induced hypohydration cases, the body composition changed when comparing the hypohydration with baseline or rehydration. One study conducted among young adults showed that individuals who were hypohydrated had higher ICW/TBW, and lower ECW and ECW/TBW than baseline values (42). Another study conducted among athletes revealed that participants seemed to be dehydrated after an Ironman triathlon compared with baseline values (75). Thus, hydration status may not impact body composition among athletes in free-living conditions. Differences in the methods used in the studies and the indices evaluating hydration status and body composition could explain the controversial results of the research mentioned above, with the current study being a cross-sectional study, whereas the others were self-controlled or randomized controlled studies.

The body water compartments of the athletes in our study were evaluated by BIA. Some studies have demonstrated the validity and reliability of the BIA in assessing the body water (60). In contrast, some studies came to the conclusions that overestimation of body fat mass and lower estimation of TBW should be considered when evaluating the body composition in health-clinical practice. The measurements of the BIA maybe impacted by factors including the nutritional status, hydration statuses, machine specifications and the technical skill (76–78). Maybe such a lack of validity could attributed to the differences of the present study and other studies, therefore, more studies should be conducted to explore the issue further.

Our findings highlight the need for tracking body composition, specifically ICW, ECW, TBW, and FFM hydration, in athletes to avoid reductions. The study had strenghs and limitations. In regard to the strengths, it was the first study investigated the correlations between water intake and body water among athletes with different total drinking fluids. Moreover, 7-day 24 h fluid intake questionnaire and the duplicate portion method were used to assess the total drinking fluids for seven consecutive days and amounts of water from all the food the participants ate for three consecutive days which would reduce the recall bias. There are limitations to this investigation to consider. The study was conducted only among male young athletes, not including females. Additionally, future studies including a larger population of different ages of adults are needed to verify our findings.

## 5 Conclusions

The present results confirmed moderate associations between water intake and body composition among athletes with habitual water intake in free-living conditions in China. Furthermore, water intake, but not hydration statuses, was found to affect body composition among athletes in free-living conditions.

## Data Availability

The datasets of this study is available from the corresponding author on reasonable request.
