# Supplementary material for: Habitual water intake impacted the body composition of young male athletes in free-living conditions: a cross-sectional study
Source: Front Sports Act Living. 2024 Oct 22;6:1458242. doi: 10.3389/fspor.2024.1458242 (PMC11534665; doi:10.3389/fspor.2024.1458242)
Supplement: Supplementary file 1 [file Table1.docx]

**Supplementary Table1** The characteristics of participants

|  | Total (*n*=109) |
| --- | --- |
| Age (y) | 20.8±1.0 |
| Height (cm) | 178.7±5.2 |
| Weight (kg) | 70.7±7.3 |
| BMI (Kg/m^2^) | 22.1±2.0 |
| skeletal muscle | 34.9±3.3 |

Note: Values are shown as the mean±standard deviation (SD). BMI: Body Mass Index.

**Supplementary Table2** The TWI, total drinking fluids and water from food among participants consuming different levels of total drinking fluids

|  | Total (*n*=109) | | |
| --- | --- | --- | --- |
|  | M | Q | % |
| Total drinking fluids | 1789 | 863 | 65.0 |
| Water | 1181 | 666 | 64.8 |
| Tea | 0 | 0 | 0.6 |
| Milk and milk products | 40 | 111 | 5.0 |
| SSBs | 469 | 424 | 27.2 |
| Sports drinks | 65 | 154 | 5.4 |
| Other SSBs | 383 | 365 | 21.8 |
| Alcohol | 0 | 0 | 2.3 |
| Others | 0 | 0 | 0.1 |
| Water from food | 955 | 472 | 35.0 |
| Staple food | 330 | 107 | 36.5 |
| Dishes | 458 | 213 | 46.7 |
| Soup | 0 | 0 | 3.3 |
| Porridge | 60 | 151 | 9.5 |
| Snacks | 9 | 64 | 4.0 |
| Total water intake | 2701 | 973 | _ |

Note: Values are shown as the median (M) and quartile ranges (Q);

**Supplementary Table3** The characteristics of 24h urine among participants consuming different levels of total drinking fluids

|  | Total (*n*=109) | |
| --- | --- | --- |
|  | M | Q |
| Volume (mL) | 850 | 408 |
| urine Osmolality (mOsm/kg) | 764 | 286 |
| (≤500 mOsm/kg, n, % ) | 17 (15.6%) | |
| Void | 3.9±1.2 | |
| Na (mmol/L) | 202 | 66 |
| K (mmol/L) | 45.21 | 12.93 |
| Cl (mmol/L) | 221 | 53 |
| USG | 1.020 | 0.007 |
| pH | 6.3 | 0.5 |

Note: Values are shown as the median (M) and quartile ranges (Q).

**Supplementary Table4** The characteristics of blood samples among participants consuming different levels of total drinking fluids

|  | Total (*n*=109) |
| --- | --- |
| Copeptin (ρmmol/L) | 1.67±0.13 |
| Testosterone (nmol/L) | 16.9±2.5 |
| Cortisol (ng/L) | 83.3±14.9 |
| Creatinine (μmmol/L) | 65.7±14.9 |
| Na (mmol/L) | 141±4 |
| K (mmol/L) | 4.30±0.66 |
| Cl (mmol/L) | 103±7 |

Note: Values are shown as the mean±standard deviation (SD).
