# Supplementary material for: Habitual water intake impacted the body composition of young male athletes in free-living conditions: a cross-sectional study
Source: Front Sports Act Living. 2024 Oct 22;6:1458242. doi: 10.3389/fspor.2024.1458242 (PMC11534665; doi:10.3389/fspor.2024.1458242)
Supplement: Supplementary file 2 [file Table2.docx]

**Supplementary Table1** The characteristics of participants

|  | LD_1_ (*n*=27) | LD_2_ (*n*=28) | LD_3_ (*n*=27) | HD (*n*=27) | Total (*n*=109) | *P* |
| --- | --- | --- | --- | --- | --- | --- |
| Age (y) | 20.4±1.1 | 20.6±1.0 | 20.9±1.2 | 20.8±1.0 | 20.8±1.0 | 0.308 |
| Height (cm) | 177.4±4.1 | 178.4±5.1 | 178.1±5.2 | 180.8±5.8 | 178.7±5.2 | 0.082 |
| Weight (kg) | 71.2±5.9 | 69.9±6.4 | 69.5±8.1 | 72.2±8.6 | 70.7±7.3 | 0.500 |
| BMI (Kg/m^2^) | 22.6±1.5 | 22.0±2.1 | 21.9±2.2 | 22.1±2.1 | 22.1±2.0 | 0.545 |
| skeletal muscle | 34.1±2.5 | 34.7±2.7 | 34.6±3.2 | 36.2±4.2 | 34.9±3.3 | 0.116 |

Note: Values are shown as the mean±standard deviation (SD). BMI: Body Mass Index.

**Supplementary Table2** The TWI, total drinking fluids and water from food among participants consuming different levels of total drinking fluids

|  | LD_1_ (*n*=27) | | | LD_2_ (*n*=28) | | | LD_3_ (*n*=27) | | | HD (*n*=27) | | | Total (*n*=109) | | |
| --- | --- | --- | --- | --- | --- | --- | --- | --- | --- | --- | --- | --- | --- | --- | --- |
|  | M | Q | % | M | Q | % | M | Q | % | M | Q | % | M | Q | % |
| Total drinking fluids | 1252 | 280 | 56.9^a^ | 1578 | 140 | 63.3^de^ | 1952 | 280 | 67.2^b^ | 2685 | 687 | 72.7^cf^ | 1789 | 863 | 65.0 |
| Water | 720 | 414 | 64.0 | 951 | 349 | 61.2 | 1370 | 255 | 64.4 | 1934 | 653 | 69.7 | 1181 | 666 | 64.8 |
| Tea | 0 | 0 | 1.0 | 0 | 0 | 0.5 | 0 | 0 | 0.7 | 0 | 0 | 0.3 | 0 | 0 | 0.6 |
| Milk and milk products | 31 | 107 | 7.4 | 33 | 104 | 3.8 | 50 | 99 | 4.4 | 50 | 196 | 4.3 | 40 | 111 | 5.0 |
| SSBs | 231 | 241 | 25.4 | 554 | 334 | 31.8 | 535 | 323 | 27.5 | 605 | 422 | 24.0 | 469 | 424 | 27.2 |
| Sports drinks | 0 | 71 | 3.3 | 71 | 139 | 5.4 | 71 | 169 | 6.1 | 141 | 193 | 6.8 | 65 | 154 | 5.4 |
| Other SSBs | 231 | 241 | 22.0 | 440 | 275 | 26.4 | 429 | 282 | 21.4 | 423 | 427 | 17.2 | 383 | 365 | 21.8 |
| Alcohol | 0 | 0 | 2.2 | 0 | 45 | 2.7 | 0 | 86 | 3.0 | 0 | 0 | 1.2 | 0 | 0 | 2.3 |
| Others | 0 | 0 | 0.0 | 0 | 0 | 0.0 | 0 | 0 | 0.1 | 0 | 0 | 0.5 | 0 | 0 | 0.1 |
| Water from food | 894 | 363 | 43.1^a^ | 946 | 381 | 36.7^de^ | 1002 | 579 | 32.8^b^ | 960 | 421 | 27.3^cf^ | 955 | 472 | 35.0 |
| Staple food | 330 | 213 | 39.4 | 322 | 92 | 35.0 | 324 | 99 | 35.6 | 363 | 113 | 36.2 | 330 | 107 | 36.5 |
| Dishes | 458 | 224 | 44.9 | 479 | 222 | 48.0 | 407 | 213 | 47.7 | 473 | 225 | 46.2 | 458 | 213 | 46.7 |
| Soup | 0 | 0 | 2.0 | 0 | 31 | 3.5 | 0 | 85 | 3.7 | 0 | 0 | 3.7 | 0 | 0 | 3.3 |
| Porridge | 56 | 179 | 10.3 | 48 | 130 | 8.5 | 43 | 146 | 8.1 | 83 | 171 | 11.1 | 60 | 151 | 9.5 |
| Snacks | 0 | 56 | 3.3 | 14 | 64 | 4.9 | 17 | 69 | 4.9 | 0 | 72 | 2.8 | 9 | 64 | 4.0 |
| Total water intake | 2133^a^ | 569 | _ | 2550^d^ | 333 | _ | 2900^b,f^ | 712 | _ | 3753^c,e^ | 864 | _ | 2701 | 973 | _ |

Note: Values are shown as the median (M) and quartile ranges (Q);

a: There was statistically significant difference between LD_1_ and LD_2_ groups, *p* < 0.05; b: There was statistically significant difference between LD_1_ and LD_3_ groups, *p* < 0.05; c: There was statistically significant difference between LD_1_ and HD groups, *p* < 0.05; d: There was statistically significant difference between LD_2_ and LD_3_ groups, *p* < 0.05; e: There was statistically significant difference between LD_2_ and HD groups, *p* < 0.05; f: There was statistically significant difference between LD_3_ and HD groups, *p* < 0.05.

%: Contributions of total drinking fluids and water from food to TWI; percentages of different fluids in total drinking fluids; proportions of water from different foods in water from food.

There were statistical significances in the amounts of TWI and total drinking fluids (*χ^2^*=77.958, *P*＜0.001; *χ^2^*=101.255, *P*＜0.001;) among the four groups, respectively; but no significant differences in the amounts of water from food (*χ^2^*=1.158, *P*=0.763). There were statistical significances in the consumption of water, SSBs, sports drinks, other SSBs and others among the four groups (*χ^2^*=68.000, *P*＜0.001; *χ^2^*=18.314, *P*＜0.001; *χ^2^*=15.260, *P*=0.002; *χ^2^*=11.434, *P*=0.010; *χ^2^*=9.148, *P*=0.027), with no significant differences were found in the volumes of milk and milk products, tea and alcohol (*χ^2^*=2.802, *P*=0.423; *χ^2^*=1.473, *P*=0.689; *χ^2^*=2.939, *P*=0.401). There were statistical significances in the contributions of total drinking fluids and water from food to TWI, respectively (*F*=23.096, *P*＜0.001; *F*=23.096, *P*＜0.001). The contributions of water, milk and milk products, tea, alcohol, other SSBs, sports drinks and SSBs in total drinking fluids did not differ significantly among the four groups (*F*=1.144, *P*=0.335; *F*=1.561, *P*=0.203; *F*=0.354, *P*=0.786; *F*=0.582, *P*=0.628; *F*=2.394, *P*=0.073; *F*=0.968, *P*=0.411; *F*=1.355, *P*=0.261), and significant differences were found in the contributions of others (*F*=2.912, *P*=0.038).

No significant differences were found in the volumes of water from staple food, dishes, soup, porridge and snacks (*χ^2^*=2.709, *P*=0.439; *χ^2^*=1.406, *P*=0.704; *χ^2^*=0.420, *P*=0.936; *χ^2^*=1.314, *P*=0.726; *χ^2^*=2.267, *P*=0.519). No significant differences were found in the contributions of staple food, dishes, soup, porridge and snacks to water from food (*F*=1.408, *P*=0.245; *F*=0.531, *P*=0.662; *F*=0.363, *P*=0.780; *F*=0.484, *P*=0.694; *F*=0.656, *P*=0.581).

**Supplementary Table3** The characteristics of 24h urine among participants consuming different levels of total drinking fluids

|  | LD_1_ (*n*=27) | | LD_2_ (*n*=28) | | LD_3_ (*n*=27) | | HD (*n*=27) | | Total (*n*=109) | |
| --- | --- | --- | --- | --- | --- | --- | --- | --- | --- | --- |
|  | M | Q | M | Q | M | Q | M | Q | M | Q |
| Volume (mL) | 711 | 386 | 726 | 534 | 876 | 281 | 938 | 513 | 850 | 408 |
| urine Osmolality (mOsm/kg) | 858 | 292 | 774 | 200 | 732 | 303 | 631 | 379 | 764 | 286 |
| (≤500 mOsm/kg, n, % ) | 2 (11.8%)^a*^ | | 1 (5.9%)^ab^ | | 4 (23.5%)^b^ | | 10 (58.8%)^b^ | | 17 (15.6%) | |
| Void | 3.8±1.2 | | 3.8±1.3 | | 3.9±1.1 | | 4.2±1.3 | | 3.9±1.2 | |
| Na (mmol/L) | 229 | 63 | 212 | 52 | 186 | 74 | 192 | 85 | 202 | 66 |
| K (mmol/L) | 45.45 | 14.59 | 48.25 | 9.76 | 42.14 | 13.99 | 41.29 | 15.00 | 45.21 | 12.93 |
| Cl (mmol/L) | 222 | 35 | 229 | 35 | 203 | 49 | 218 | 83 | 221 | 53 |
| USG | 1.020 | 0.010 | 1.022 | 0.007 | 1.020 | 0.007 | 1.020 | 0.008 | 1.020 | 0.007 |
| pH | 6.7 | 0.7 | 6.2 | 0.6 | 6.3 | 0.5 | 6.3 | 0.6 | 6.3 | 0.5 |

Note: Values are shown as the median (M) and quartile ranges (Q); ^*^χ^2^=20.274, *P*=0.002;

a: There was statistically significant difference between LD_1_ and LD_2_ groups, *p* < 0.05; b: There was statistically significant difference between LD_1_ and LD_3_ groups, *p* < 0.05; c: There was statistically significant difference between LD_1_ and HD groups, *p* < 0.05; d: There was statistically significant difference between LD_2_ and LD_3_ groups, *p* < 0.05; e: There was statistically significant difference between LD_2_ and HD groups, *p* < 0.05; f: There was statistically significant difference between LD_3_ and HD groups, *p* < 0.05.

Significant differences were found in the volume, osmolality, the concentrations of Na, and Cl (χ^2^=9.141, *P*=0.027; χ^2^=12.831, *P*=0.005; χ^2^=9.900, *P*=0.019), but no significant differences were found in the voids, K, USG and pH among the four groups (*F*=0.567, *P*=0.638; χ^2^=6.627, *P*=0.085; χ^2^=2.945, *P*=0.400; χ^2^=6.148, *P*=0.105).

**Supplementary Table4** The characteristics of blood samples among participants consuming different levels of total drinking fluids

|  | LD_1_ (*n*=27) | LD_2_ (*n*=28) | LD_3_ (*n*=27) | HD (*n*=27) | Total (*n*=109) | *P* |
| --- | --- | --- | --- | --- | --- | --- |
| Copeptin (ρmmol/L) | 1.68±0.14 | 1.66±0.15 | 1.65±0.12 | 1.68±0.10 | 1.67±0.13 | 0.776 |
| Testosterone (nmol/L) | 17.4±2.6 | 17.5±2.6 | 16.4±2.2 | 16.4±2.2 | 16.9±2.5 | 0.177 |
| Cortisol (ng/L) | 80.9±11.4 | 81.4±16.9 | 84.1±14.9 | 96.9±15.7 | 83.3±14.9 | 0.425 |
| Creatinine (μmmol/L) | 65.5±14.2 | 65.2±16.8 | 65.5±15.0 | 66.8±14.1 | 65.7±14.9 | 0.980 |
| Na (mmol/L) | 141±3 | 142±3 | 141±3 | 140±4 | 141±4 | 0.327 |
| K (mmol/L) | 4.06±0.51 | 4.20±0.64 | 4.31±0.63 | 4.63±0.73^c^ | 4.30±0.66 | 0.010 |
| Cl (mmol/L) | 104±8 | 102±6 | 104±7 | 102±7 | 103±7 | 0.534 |

Note: Values are shown as the mean±standard deviation (SD); a: There was statistically significant difference between LD_1_ and LD_2_ groups, *p* < 0.05; b: There was statistically significant difference between LD_1_ and LD_3_ groups, *p* < 0.05; c: There was statistically significant difference between LD_1_ and HD groups, *p* < 0.05; d: There was statistically significant difference between LD_2_ and LD_3_ groups, *p* < 0.05; e: There was statistically significant difference between LD_2_ and HD groups, *p* < 0.05; f: There was statistically significant difference between LD_3_ and HD groups, *p* < 0.05.

No significant differences were found in the concentrations of copeptin, testosterone, cortisol, creatinine, Na and Cl among the four groups (*F*=0.368, *P*=0.776; *F*=1.676, *P*=0.177; *F*=0.938, *P*=0.425; *F*=0.061, *P*=0.980; *F*=0.734, *P*=0.534; *F*=1.163, *P*=0.327), except the concentrations of K (*F*=3.965, *P*=0.010).
